# Characterization of variably protease-sensitive prionopathy by capillary electrophoresis

**DOI:** 10.1038/s41598-024-79217-1

**Published:** 2024-11-13

**Authors:** Jennifer Myskiw, Ben A. Bailey-Elkin, Kristen Avery, Marcelo A. Barria, Diane L. Ritchie, Mark L. Cohen, Brian S. Appleby, Stephanie A Booth

**Affiliations:** 1 Mycobacteriology, Vector-borne and Prion Diseases Division, Public Health Agency of Canada, National Microbiology Laboratory, Winnipeg, MB Canada; 2 Department of Medical Microbiology and Infectious Diseases, Faculty of Health Sciences, University of Manitoba, Winnipeg, MB Canada; 3 National CJD Research and Surveillance Unit, Centre for Clinical Brain Sciences, University of Edinburgh, Edinburgh, UK; 4 National Prion Disease Pathology Surveillance Center, Case Western Reserve University, Cleveland, OH USA

**Keywords:** Diseases of the nervous system, Clinical microbiology, Prions

## Abstract

Variably Protease Sensitive Prionopathy (VPSPr) is a rare human prion disease that, like Creutzfeldt-Jakob disease (CJD), results in the deposition of abnormally folded prion protein aggregates in the brain and is ultimately fatal. Neuropathology and clinical features of VPSPr are heterogeneous. However, the key discriminating feature is the relative sensitivity of the pathological prion protein to proteinase digestion compared to that typically seen in other human prion cases. Three major fragments of 23, 17 and 7 kDa are characteristic of the disease following digestion with proteinase K. We recently reported the utility of the highly adaptive and reproducible ProteinSimple™ capillary electrophoresis (CE) system to perform protein separation of PK digested prion protein in CJD. Consequently, we explored capillary-based electrophoresis (CE) technology as a sensitive method to detect and characterize VPSPr in a cohort of 29 cases. The unique 7 kDa fragment has high intensity, particularly in cases with the codon 129 VV genotype, but can be missed by regular Western blotting due to the small size. However, this fragment is readily detected by CE in all cases. In addition, the flexibility of CE produced highly reproducible, semi-quantitative data for determining relative proteinase K sensitivity and epitope mapping of representative cases from each codon 129 genotype (VV, MV and MM).

## Introduction

Human prion diseases are fatal neurodegenerative disorders caused by misfolded cellular prion proteins (PrP^C^) that aggregate to form brain deposits, resulting in spongiform vacuolation and neuronal loss. The most prevalent human prion disease is Creutzfeldt-Jakob disease (CJD), with a worldwide annual incidence of 1–2 cases per million individuals^1^. Most commonly, CJD occurs sporadically, although genetic and acquired forms also occur. In disease, PrP^C^, a primarily α-helical prion protein, spontaneously misfolds, taking on a pathological conformation highly enriched in β-sheet secondary structure, PrP^Sc^, that can self-propagate^2,3^. Uniquely, the C-terminus of PrP^Sc^ is highly resistant to proteases, and this characteristic is exploited for diagnosing CJD, as digestion with proteinase K (PK) leads to distinctive fragment sizes based on tertiary structure and glycosylation site occupancy. A molecular classification scheme for CJD that considers the apparent molecular weight of the PK-resistant unglycosylated fragment of PrP^Sc^ (21 or 19 kDa) and the methionine (M)/valine (V) polymorphism at codon-129 (MM, VV or MV) on the prion protein gene (PRNP) has been adopted worldwide^4–6^. The resulting six subtypes largely correspond with disease phenotypes in sporadic CJD. However, clinicopathological features may not always correlate, and variations in PK-resistant fragment sizes and glycosylation are occasionally identified. Evidently, atypical cases representing alternative prion strains or unique subtypes exist^7–11^.

One such novel sporadic human prion disease is variably protease-sensitive prionopathy (VPSPr) that was first described in 2008, and cases have since been identified in USA^10^, UK^12–16^, the Netherlands^17^, Spain^18^ and Canada^19^. This disease is typified by an extended disease course and the presence of micro plaques in the brain as well as distinctive biochemistry, where PrP^Sc^ is more susceptible to proteolytic treatment^10,17,20^. A hallmark low molecular weight (7-8 kDa) band is detected by Western blot in VPSPr cases that is similar to that seen in the genetic prionopathy Gerstmann-Straussler-Scheinker syndrome (GSS); however, no mutations have been associated to VPSPr^10^. VPSPr is also unique from CJD in that di-glycosylated VPSPr PrP^C^ does not convert to PrP^Sc^, and therefore, di-glycosylated fragments are notably absent on Western blots in these cases^20–22^. Subsequent studies confirmed the transmissibility of VPSPr in humanized mice, albeit at reduced efficiency compared to CJD, supporting the direct role of VPSPr-derived PrP^Sc^ in disease pathology^23^.

We previously described a capillary-based electrophoresis system capable of high-throughput PrP^Sc^ characterization in CJD cases, which demonstrated improved sensitivity compared to traditional Western blot methods and an enhanced capacity to detect cases of VPSPr^19^. Here, we expand on this work to illustrate the utility of capillary electrophoresis methods in characterizing VPSPr. Using a large VPSPr cohort (29 cases), we have identified and characterized the biochemical profile of VPSPr by capillary electrophoresis across all codon 129 genotypes and indirectly investigated the structure of VPSPr-derived PrP^Sc^ by epitope mapping of select cases.

## Results

### Characterization of a cohort of 29 cases of VPSPr human sporadic prionopathies

**Fig. 1:**
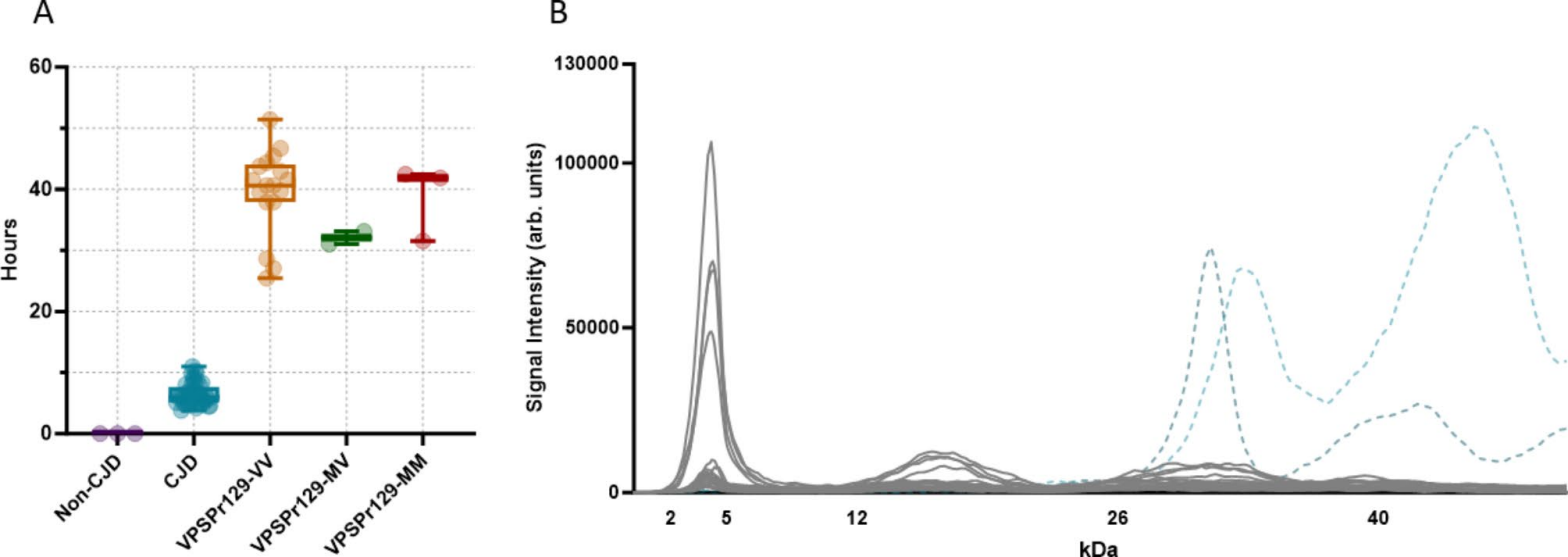
Seeding lag phase and electrophoretogram of VPSPr PrPSc immunoprobed with antibody 3F4. (**A**) Seeding lag phases of PrPSc from non-CJD, sCJD, and VPSPr (all genotypes) 10% (w/v) brain homogenates at a 10^− 4^ dilution were seeded with recombinant full-length hamster PrPC using the real-time quaking-induced conversion (RT-QuIC) assay. Time from the assay start time to when a pre-defined amyloid formation threshold was reached (lag phase time) was calculated and plotted along with bars showing the min–max values. sCJD (n=33) displayed with a lag phase time ranging from 5 to 11 hours. VPSPr (n=20) displayed a longer lag phase, ranging from 25 to over 50 hours to reach the threshold. (**B**) Overlay of electrophoretograms from 29 VPSPr Cases (grey) and individual representative type 1 and 2 CJD cases (light and dark blue dotted lines, respectively) PK-digested (25 µg/ml) and immunoprobed with antibody 3F4 using the CE immunoassay.

A cohort of 29 VPSPr cases from the United States of America (*n* = 20), the United Kingdom (*n* = 7), and Canada (*n* = 2) were used in this study. All VPSPr cases were diagnosed and defined by genotype in their respective countries before being sent to our laboratory for this study, Table 1. We confirmed that all cases contained prion seeding activity by real-time quaking-induced conversion (RT-QuIC) assays using diluted brain homogenate (10^− 4^). VPSPr is associated with an extended disease duration in patients and following transmission to animal models^10,23^. We, therefore, compared the lag phase (the time from the start of the assay to the point at which seed-dependent amyloid formation reached a threshold for detection) with equivalent data from sporadic CJD patients. Seeding was uniformly slow in all 29 VPSPr patients tested (25–50 h) versus 5–11 h for sCJD, Fig. 1A.

**Fig. 2:**
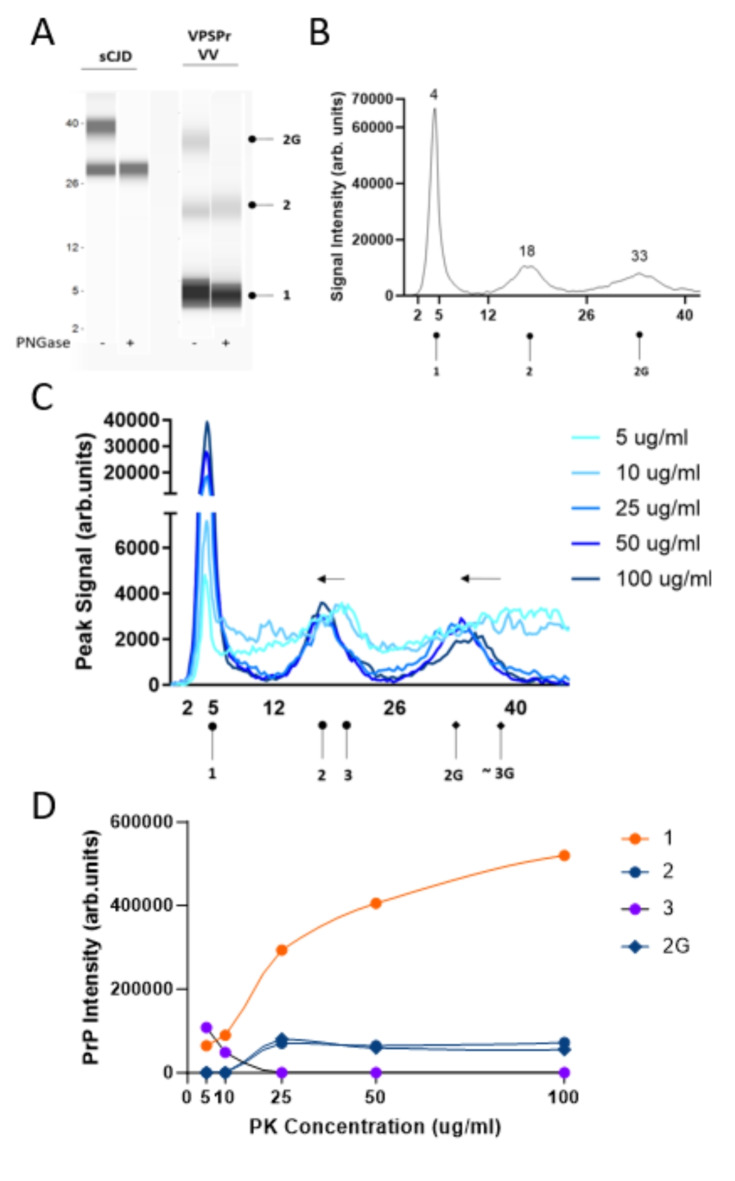
Electrophoretic profiles of VPSPr129-VV fragments and their sensitivity to proteinase-K digestion. (**A**) Mock “western blot view” of sCJD and VPSPr129-VV digested with 25 µg/ml of PK treated with or without PNGase using the CE assay. Circle heads and labels are used to annotate VPSPr fragments: “1” is the C-terminal truncated unglycosylated fragment, “2” is the larger C-terminal truncated unglycosylated fragment, “2G” is the monoglycosylated form of “2”, “3” is the full-length unglycosylated fragment (not detected at 25 µg/ml, shown in Panel C), and “3G” is the monoglycosylated form of “3” (not detected at 25 µg/ml, shown in Panel C) (**B**) Electrophoretogram of VPSPr129-VV digested with 25 µg/ml of PK (**C**) Electrophoretogram of PK-titration assay of a VPSPr129-VV case. The arrows demonstrate the shift from a higher molecular weight to a lower molecular weight due to the truncation of fragments as PK concentrations increase. While we did observe a rough signal where the band “3G” was anticipated to be located, the signal was too faint to indicate an exact molecular weight. (**D**) Graph showing the PrP signal intensity change of each VPSPr fragment throughout the PK titration assay.

**Fig. 3:**
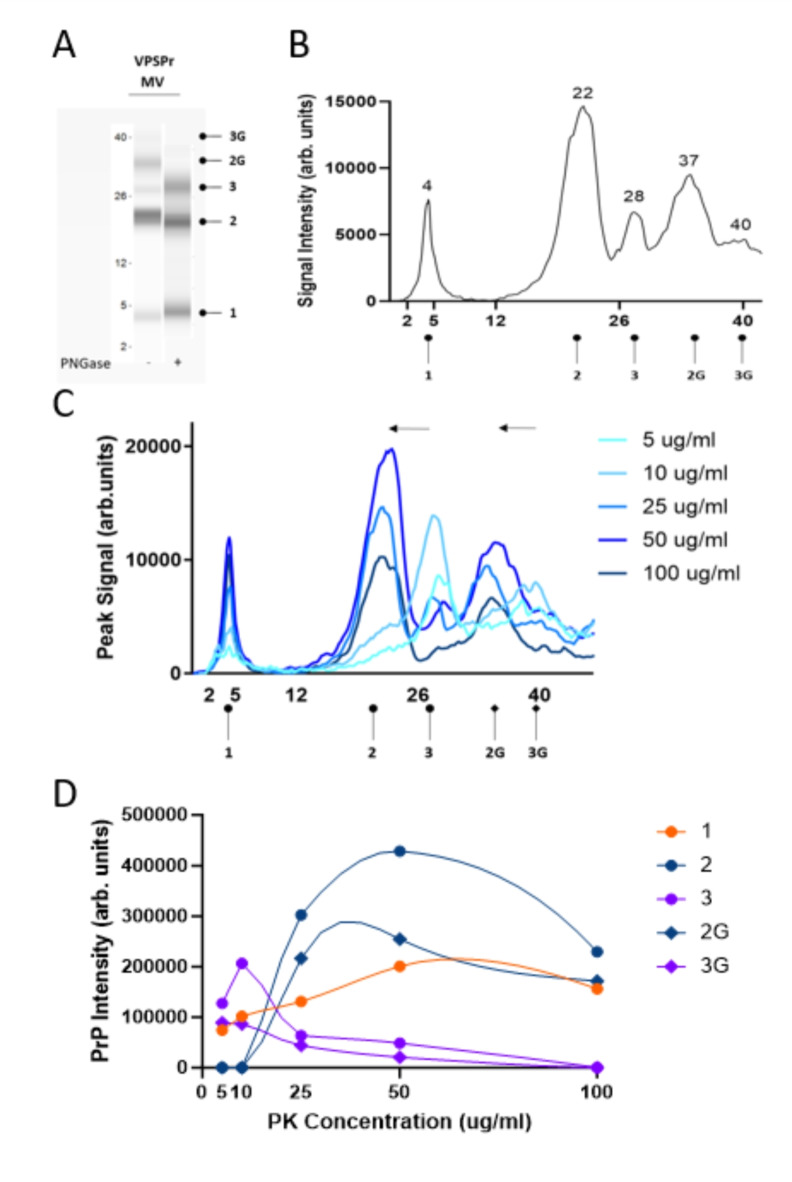
Electrophoretic profiles of VPSPr129-MV fragments and their sensitivity to proteinase-K digestion. (**A**) Mock “western blot view” of VPSPr129-MV digested with 25 µg/ml of PK treated with or without PNGase using the CE assay. Fragments are labelled according to convention described in Figure 2. (**B**) Electrophoretogram of VPSPr129-MV digested with 25 µg/ml of PK (**C**) Electrophoretogram of PK-titration assay of a VPSPr129-MV case. The arrows demonstrate the shift from a higher molecular weight to a lower molecular weight due to the truncation of fragments as PK concentrations increase (3 → 2 and 3G → 2G). (**D**) Graph showing the PrP signal intensity change of each VPSPr fragment throughout the PK titration assay.

**Fig. 4:**
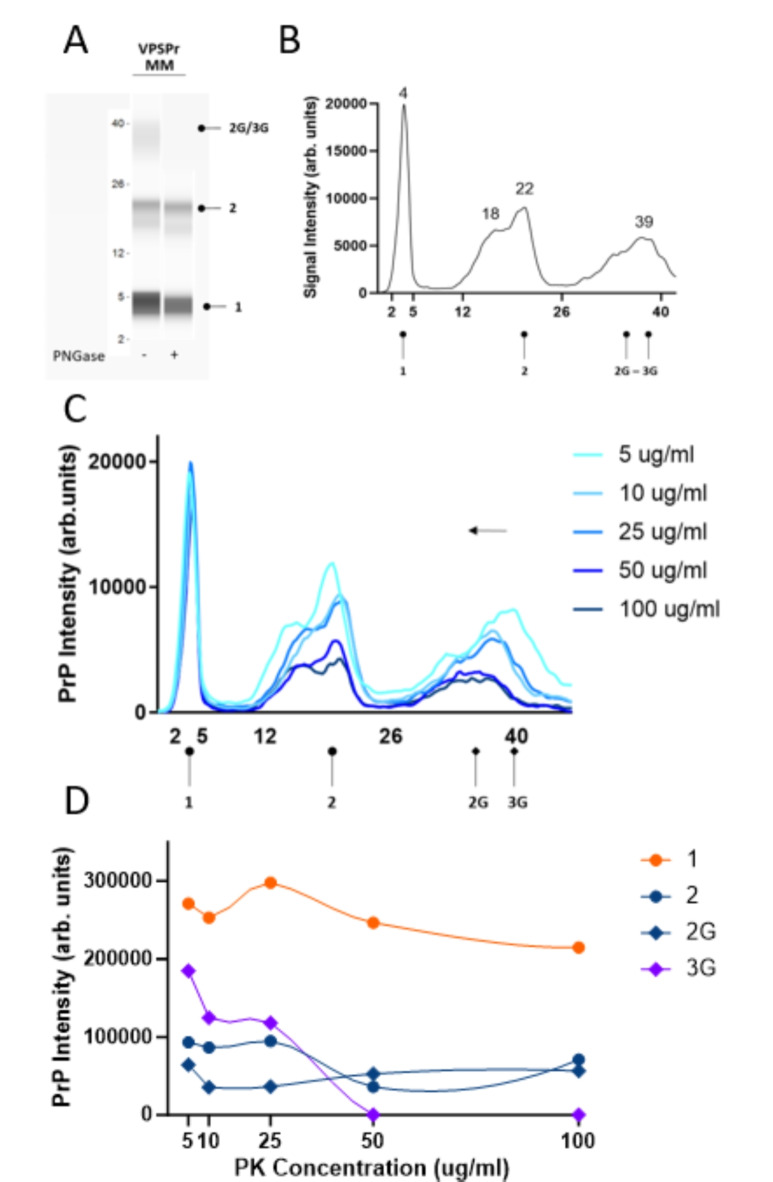
Electrophoretic profiles of VPSPr129-MM fragments and their sensitivity to proteinase-K digestion. (**A**) Mock “western blot view” of VPSPr129-MM digested with 25 µg/ml of PK treated with or without PNGase using the CE assay. Fragments are labelled according to convention described in Figure 2. (**B**) Electrophoretogram of VPSPr129-MM digested with 25 µg/ml of PK (**C**) Electrophoretogram of PK-titration assay of a VPSPr129-MM case. The arrows demonstrate the shift from a higher molecular weight to a lower molecular weight due to the truncation of fragments as PK concentrations increase (3G → 2G) (**D**) Graph showing the PrP signal intensity change of each VPSPr fragment throughout the PK titration assay.

We previously described the capillary electrophoresis assay as a convenient and accurate way to detect the low molecular weight 7 kDa PK-resistant fragment that is stereotypical of VPSPr cases^19^. As previously noted, the relative molecular weights of fragments determined by the CE immunoassay are not directly comparable to those determined by western immunoblotting^19,24–26^. However, bands corresponding to fragments confirmed by western blot can be deduced by observing trends in fragmentation patterns and establishing the glycosylation state of observed PK-resistant species. Phosphotungstate anion (PTA)-precipitated and PK-digested brain homogenates from 29 VPSPr cases were analyzed using antibody 3F4, which recognizes amino acids 106–112 of human PrP. The small fragment with an apparent molecular weight of 4 kDa on capillary electrophoresis (corresponding to the 7 kDa fragment by western blot) was consistently detected in all 29 cases, Fig. 1B. This was independent of the codon 129 genotype, of which 23 cases were VV, 3 were MV, and 3 were MM. Treatment of PK-digested samples with PNGase confirmed the identity of higher molecular weight mono- and unglycosylated species, Figs. 2A, 3A, 4A.

**Table 1:** Cohort codon 129 genotype and disease duration.

Case ID Number	Originating region	Codon129 genotype	Disease duration (months)
1	Canada	VV	45
2	Canada	VV	14
3	UK	VV	41
4	UK	VV	~ 90
5	UK	VV	~ 5
6	UK	MV	12
7	UK	VV	41
8	UK	VV	26
9	UK	VV	38
10	USA	MV	24
11	USA	VV	48
12	USA	MV	8
13	USA	VV	11
14	USA	VV	15
15	USA	VV	21
16	USA	VV	6
17	USA	MM	45
18	USA	VV	8
19	USA	MM	30
20	USA	VV	15
21	USA	VV	9
22	USA	MM	Unknown
23	USA	VV	17
24	USA	VV	14
25	USA	VV	8
26	USA	VV	10
27	USA	VV	9
28	USA	VV	Unknown
29	USA	VV	Unknown

### Characterization of VPSPr fragments according to genotype* VPSPr129-VV*

VPSPr129-VV cases presented with three bands at apparent molecular weights by CE of 30-33 kDa, 18 kDa, and 4 kDa when probed with antibody 1E4, corresponding to N-terminal truncated monoglycosylated and unglycosylated fragments, and the smallest truncated unglycosylated fragment at 4 kDa, Fig. 2A,B. The truncated 1E4 reactive mono- and un-glycosylated fragments are faint or absent in the VPSPr129-VV cases when treated with a final PK concentration of 25 µg/ml^10,21^. To more carefully assess the protease sensitivity of VPSPr129-VV fragments, titration assays were performed by treating PTA-precipitated brain homogenate with PK at final concentrations ranging from 5 µg/ml to 100 µg/ml and, immunoprobing with 1E4, Fig. 2C. The 4 kDa band increased in intensity as the PK concentration increased. The higher molecular weight peaks at 18 and 33 kDa, representing un- and mono-glycosylated truncated species, respectively, were undetectable until PK concentrations reached 25 µg/ml, where the signal intensity remained relatively constant as PK concentration increased. An additional faint peak at 20 kDa was detectable at very low PK concentrations before becoming undetectable at 25 µg/ml. It is likely this PK-sensitive 20 kDa fragment represents the full-length form of the unglycosylated VPSPr fragment before shifting to the lower molecular weight seen at 17 kDa, with a complete shift to the truncated version at 25 µg/ml of PK, Fig. 2D.

### VPSPr129-MV

VPSPr129-MV cases presented with 5 peaks on CE at 40 kDa, 37 kDa, 28 kDa, 22 kDa and 4 kDa corresponding to full-length and truncated monoglycosylated, full-length and truncated unglycosylated and the smallest truncated fragment, Fig. 3A,B. The shift to N-terminal truncated fragments derived from the GPI-anchored (full-length) form is evident in the case of VPSPr129-MV, Fig. 3C. At PK concentrations between 25 and 50 µg/ml, the bands previously seen at 40 kDa (monoglycosylated) and 28 kDa (unglycosylated) are replaced with bands at 37 and 22 kDa, respectively, and like the trend seen in the VPSPr129-VV sample, the 4 kDa signal intensifies slightly as the PK concentration increases, reaching its maximum intensity at 50 µg/ml of PK, Fig. 3D.

### VPSPr129-MM

PK digestion of VPSPr129-MM cases produced 4 bands on CE at 37 kDa, 21 kDa, 18 kDa and 4 kDa, Fig. 4A,B. As previously described, the CE assay cannot discriminate between bands within a 1–2 kDa range of one another and instead will present as a wider peak^19^. Notably, the band at 37 kDa, corresponding to the monoglycosylated fragment that resolves at 23–26 kDa on traditional western blotting, presents with a broad signal and jagged peak, which may reflect multiple fragments that are close in size or heterogeneous glycan chain lengths. Similar to VPSPr129-MV, these fragments migrated slightly slower than those detected in VPSPr129-VV cases^20^.

PK titration experiments with VPRSr129-MM revealed that, unlike the other genotypes, the intensity of the 4 kDa peak remains relatively constant throughout the PK titration, Fig. 4C. A shift from the mono-glycosylated fragment at 40 kDa to the truncated 37 kDa form is apparent, with the 40 kDa band becoming nearly undetectable at the low PK concentration of 10 µg/ml. Interestingly, throughout the range of PK concentrations, two partially overlapping peaks are detected at 18 and 22 kDa, which both decrease in intensity as PK concentration is increased. Overall, in agreement with other findings, VPSPr129-MM appears to be the most resistant to PK degradation ^11^.

### Epitope mapping of VPSPr using capillary electrophoresis

**Fig. 5:**
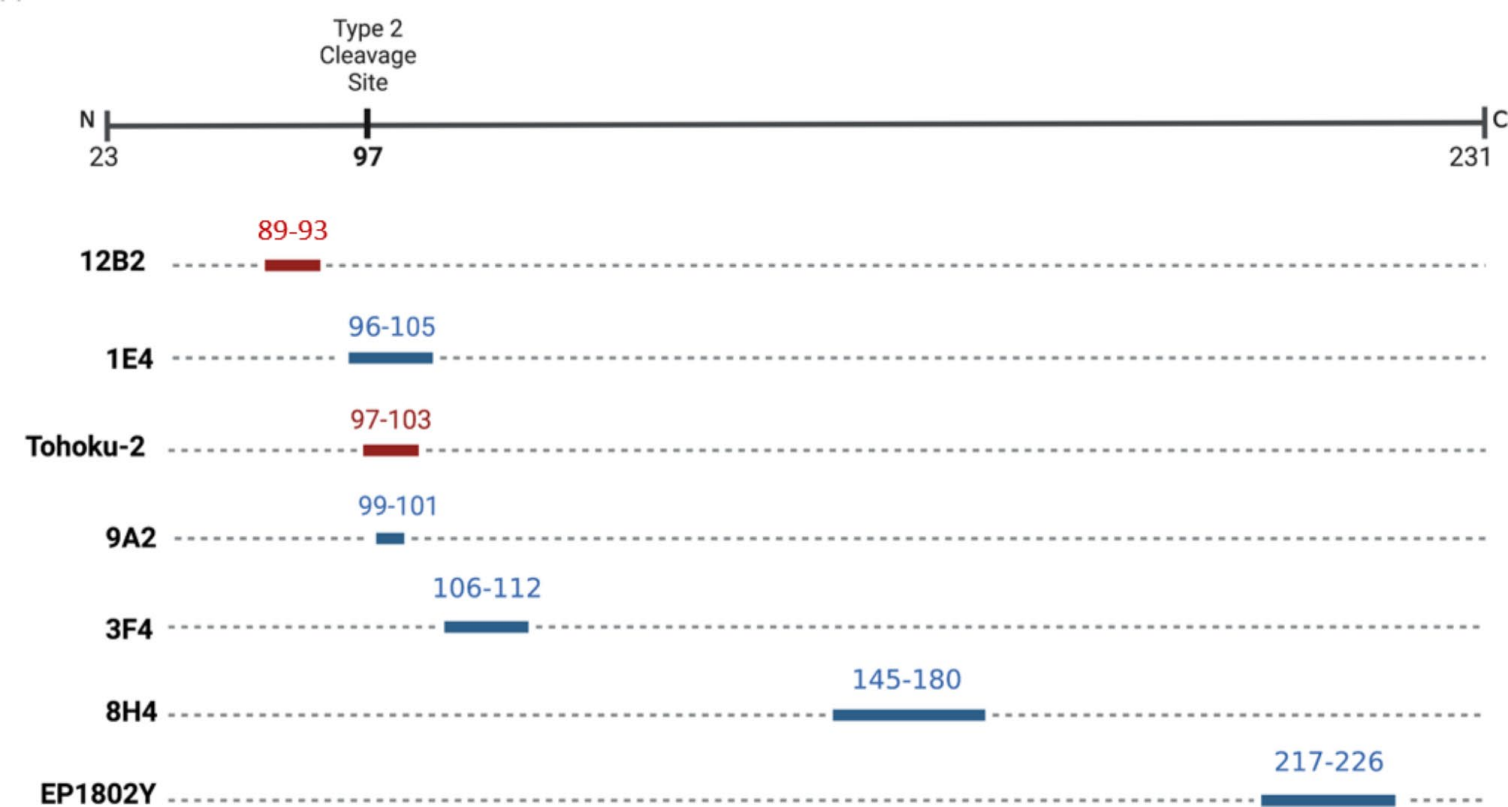
PrP-Specific Antibody Epitope Binding Regions to the Human Prion Protein. Antibodies recognizing PrP epitopes depicted in red are sCJD type-specific, 12B2 is type-1 CJD specific^47^ and Tohoku-2 is type-2 CJD specific^48^. Antibodies recognizing epitopes depicted in blue have a binding affinity to both Type 1 and 2 CJD.

**Fig. 6:**
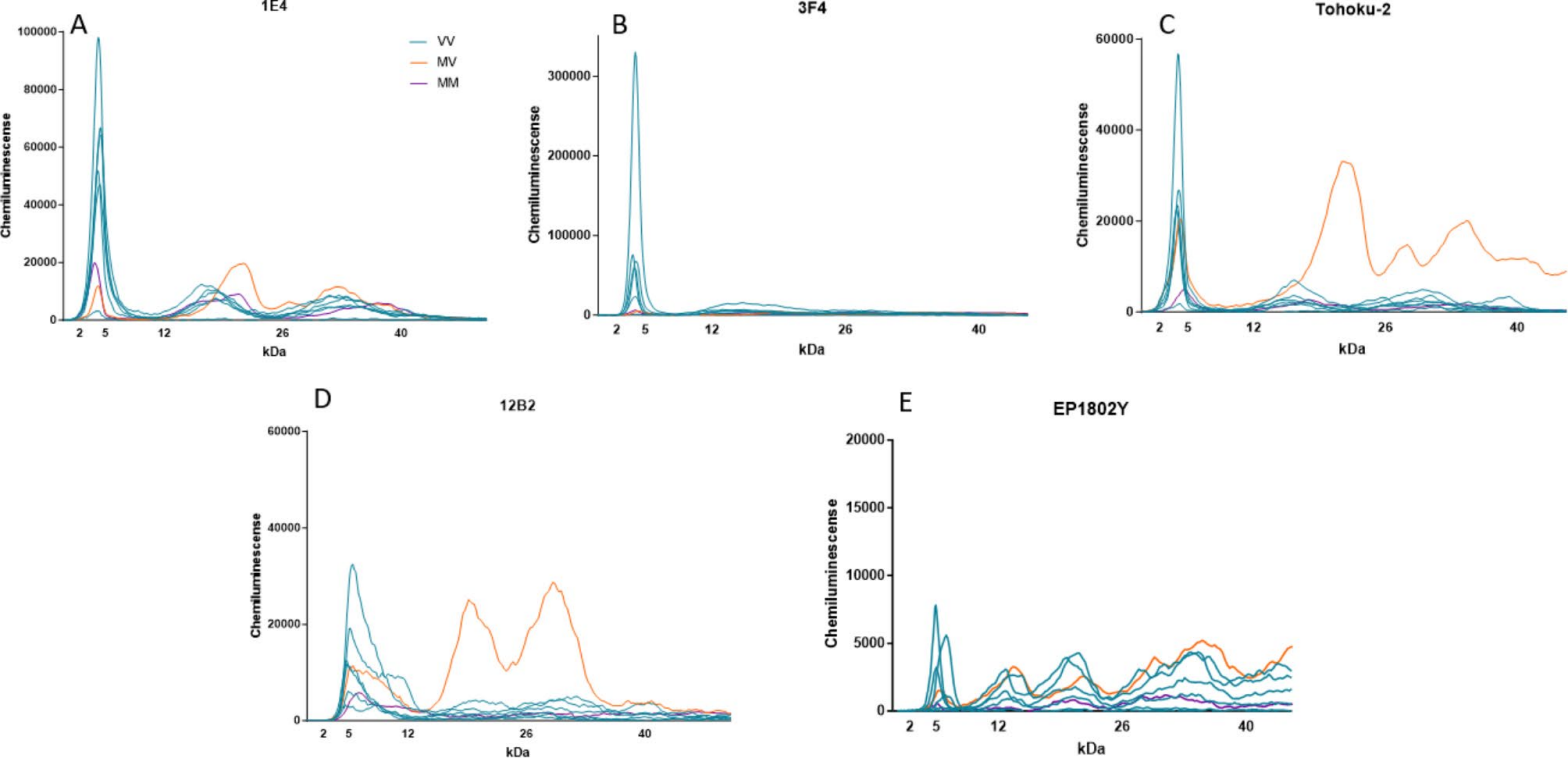
Epitope Mapping of VPSPr Cases with PrP-specific Antibodies. VPSPr129-VV, -MV and -MM cases were PTA precipitated and PK digested at a final concentration of 50 µg/ml before being probed with PrP-specific antibodies using the CE assay. Electrophoretogram data was exported and graphed on Graphpad Prism to provide signal overlays (orange MV, blue VV, and purple MM) (**A**) 1E4 (residues 96-105), (**B**) 3F4 (residues 106-112), (**C**) Tohoku-2 (residues 97-103), (**D**) 12B2 (residues 89-93), and (**E**) EP1802Y (residues 217-226).

To characterize the PrP^Sc^ species present in VPSPr cases, we measured the electrophoretic profiles of a smaller group of 8 VPSPr cases for which sufficient tissue was available (6 VV129; 1 MV129, and 1 MM129) using a panel of antibodies, Fig. 5. Following PK digestion (50 µg/ml), antibodies 1E4, 3F4, and Tohoku-2 showed similar fragment migration patterns. However, the relative proportions of each fragment detected differ, which may be due to differences in antibody binding affinities or relative abundances of PK-digested fragments Fig. 6. Antibody 3F4 bound preferentially to VPSPr129-VV cases with barely any detection of the other fragments from the VPSPr129-MV and VPSPr129-MM cases. Antibodies 1E4 and Tohoku-2 have epitopes beginning at amino acids 96 and 97, respectively; therefore, it is unsurprising that these antibodies behave similarly. Notably, all three antibodies universally detected the stereotypical 4 kDa fragment. Importantly, Tohoku-2 recognizes a PrP epitope beginning precisely at residue Ser97^27^, which is exposed following PK digestion of typical type 2 sCJD cases, suggesting that PrP^Sc^ of VPSPr adopts conformations that at least partially resemble those present in type-2 sCJD.

When probed with antibody 12B2, which recognizes residues 89–93, the 4 kDa bands observed with 1E4, 3F4 and Tohoku-2 are replaced with a slightly larger species at 6–7 kDa for all VPSPr genotypes, Fig. 6D. Since this species does not cross-react with 1E4, 3F4 and Tohoku-2 antibodies, it is likely that the N-terminal region of the 6 kDa fragment retains secondary structure during electrophoresis and occludes the 1E4/3F4/Tohoku-2 epitopes while retaining reactivity towards 12B2.

To probe the C-terminal region of VPSPr-derived PrP^Sc^, samples were also analyzed using antibody EP1802Y, Fig. 6E. VPSPr129-VV cases probed with EP1802Y showed bands at 5 kDa, 11 kDa, 20 kDa, 27 kDa, and 37 kDa. This antibody binds to an additional fragment at 11 kDa (corresponding to the 12/13 kDa fragment observed with western blotting^21,22^). The VPSPr129-MV and -MM cases exhibited similar binding profiles; however, in the MV case, the 20 kDa fragment seen in the VV case was replaced by a slightly larger fragment at 22/23 kDa. EP1802Y binds all C-terminal VPSPr fragments and is therefore available to bind to the C-terminal fragment (cleavage site of a.a 158), that lacks the N-terminal epitopes the other antibodies probe for^22^. We also probed VPSPr cases with antibody 8H4, which recognizes an epitope central in the prion protein, binding at 145–180^28^. No signal was detected with this antibody in any of the VPSPr cases (data not shown). 8H4 is not commonly used to probe human prion diseases, and it is therefore possible that this antibody has a poor affinity for human prion diseases altogether. Another possible reason as to why we did not observe a signal with 8H4, is that VPSPr PrP^Sc^ may retain conformational properties that prevent the epitope region of this antibody from binding.

### Protease resistance profiles of 12B2-binding PrPSc fragments

Our epitope mapping studies using antibody 12B2 identified a small 6 kDa molecular weight fragment in all VPSPr genotypes, which, to our knowledge, has not been identified in previous studies. To further investigate these findings, we performed PK-titration assays on representative cases of VPSPr from each genotype using antibody 12B2.

**Fig. 7:**
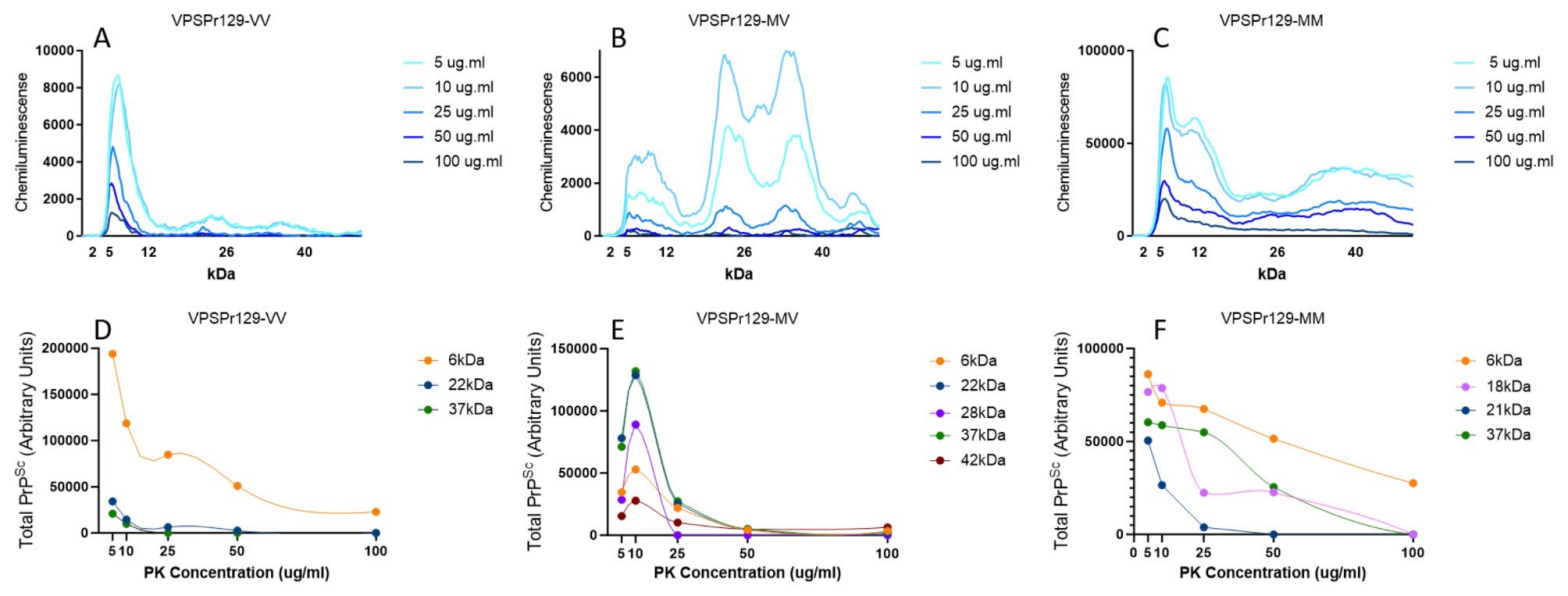
Analysis from PK-Titration of PrPSc Fragments from VPSPr129-VV, -MV, and -MM Genotypes Immunoprobed with PrP-Antibody 12B2. Equal concentrations of PTA-precipitated 10% brain homogenate were treated with PK concentrations ranging from 5 µg/ml to 100 µg/ml followed by capillary-electrophoresis to analyze conformational stability of the different VPSPr fragments from (**A**) VPSPr129-VV, (**B**) VPSPr129-MV and (**C**) VPSPr-MM. (**D**), (**E**), & (**C**) Signal intensity changes for each VPSPr fragment was evaluated for each genotype.

**Fig. 8:**
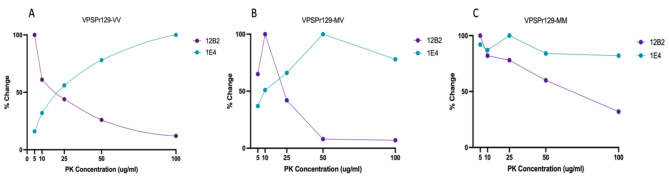
Rate of Change During PK-Titration of Low Molecular Weight VPSPr Fragments Bound to PrP-Specific 1E4 and 12B2. Rates of change were calculated for the smallest VPSPr fragment for (**A**) VPSPr129-VV (**B**) VPSPr129-MV and (**C**) VPSPr129-MM. Each graph shows the rates of change when the smallest molecular weight VPSPr fragment was immunoprobed with 1E4 (residues 96–106) and 12B2 (89–93) for a single representative sample.

Overall, we found the fragments with in-tact epitopes binding to 12B2 were highly sensitive to PK digestion, with minimal detection of VPSPr fragments at 100 µg/ml of PK for all genotypes, Fig. 7. This differs drastically from the PK digestion pattern observed in the VPSPr fragments using antibody 1E4. This difference is most pronounced in VPSPr129-VV cases when analyzing the lowest molecular weight fragments (4 kDa for 1E4 and 6 kDa for 12B2). The rates at which the 4 kDa intensity increases as PK intensifies and the rate at which the 6 kDa intensity decreases as PK concentrations increase are inversely associated with one another, Fig. 8A.

Interestingly, this association appears to be genotype-specific. The VPSPr129-MV case appeared to behave similarly to the rates of change observed with VPSPr129-VV. However, in VPSPr129-MV, both low molecular weight fragments increased at low PK concentrations before displaying an inverse relationship, Fig. 8B. Conversely, the VPSPr129-MM case showed nearly identical rates of change of the lowest molecular weight fragments (4 kDa and 6 kDa), Fig. 8C. The inverse association of these low molecular weight fragments in VPSPr129-VV and MV cases suggests that in these genotypes the 6 kDa fragment with epitope availability to 12B2 is digested by PK (N-terminal digestion), resulting in a secondary structure change that exposes the 1E4 epitope in a truncated version at 4 kDa. The 12B2-reactive epitope of VPSPr, therefore, appears to exist transiently and is rapidly digested in the presence of PK.

## Discussion

Classification of proteinase-K-resistant prion proteins through western blotting is the gold standard assay for providing diagnostic information in post-mortem CJD cases. We previously evaluated the utility of a capillary-electrophoresis system to classify CJD post-mortem and found the assay accurately described CJD cases with remarkable sensitivity^19^. The benefits of this assay include a short preparation and running time (~ 4 h total), a small volume of sample required for analysis (maximum of 3 µl sample per capillary), and a semi-quantitative read-out allowing for a reproducible, comparative analysis across samples^29^. While western blotting and capillary electrophoresis both involve the immunodetection of denatured proteins separated by size, key differences may affect the appearance of banding patterns. In western blotting, an abundance of protein is loaded into each well of a gel and probed with a small concentration of antibodies to avoid over-saturation of signal on the membrane. The CE assay is the exact opposite, where a minute amount of sample is loaded into a capillary, along with an excess of antibody to probe the samples. These differences will alter the kinetics of immunodetection, with signal detection more representative of the amount of protein loaded rather than the relative affinity of each antibody as with Western blotting. Therefore, while it is expected that both techniques should identify the same fragments, the relative intensities of each fragment may appear different with each methodology. Given the high sensitivity of the CE assay, proteins and peptides at low concentrations may be detected more readily than by Western blot. Each assay has advantages and disadvantages. Whilst CE can provide semi-quantitative data with greater consistency than the western blot, the lack of resolution between proteins that are very close in molecular weight can be a caveat to the CE method^29–32^.

VPSPr was first reported in 2008 and is the latest human prion disease to be identified. However, given its rarity, clinical and histopathologic heterogeneity, and the prion protein’s unusual sensitivity to protease digestion, the detection and characterization of this disease is challenging. VPSPr can mimic many other neurodegenerative diseases, and a diagnosis requires post-mortem immunohistochemistry and biochemical analysis of the pathogenic prion protein (PrP^Sc^) through western blotting^16,33^. However, traditional western blotting techniques that can be used to characterize other human prion diseases are inadequate to study VPSPr PrP^Sc^, and stringent conditions are typically applied^15,20,21^. This is primarily due to its PrP^Sc^ sensitivity to protease-K and the small size of its fragments (< 25 kDa on western blot). It is, therefore, expected that VPSPr cases have been underdiagnosed in the past^20,34^. Given the sensitivity of the CE assay, we explored the utility of this assay to characterize a large cohort of VPSPr cases from multiple centers with this new methodology.

To supplement the characterization of VPSPr cases in this study using CE, we first evaluated the seeding lag phases of all cases. Consistent with previous studies^15,21,35^, the lag phases of PrP^Sc^ from VPSPr patients is slower than that observed with sCJD PrP^Sc^. The slow PrP^Sc^ seeding observed in VPSPr cases support the theory that VPSPr prions represent an “incompetent” prion strain in humans and that the relatively slow aggregation results in extended disease durations seen in patients^36^. Furthermore, previous research has shown inefficient VPSPr transmission to transgenic mice and bank voles, which may be due to the inability or reduced capacity of VPSPr prions to seed aggregation in a new host^23,37^.

When analyzing VPSPr using the CE assay, VPSPr fragments were consistently detected, demonstrating its robustness in this context. Notably, the smallest VPSPr fragment at 4 kDa (7 kDa on western blot) was prominently detected in all cases, regardless of PRNP genotype or PK concentration. Also, the 4 kDa fragment was easily detectable with the antibody 3F4 in all samples, which contrasts with previous reports using western blotting techniques, where fragments were barely detectable or undetectable with the same antibody^21^. This underscores one of the key advantages of CE detection, as the excess of the 3F4 antibody applied to the capillary enhances the detection of the small fragment, despite its lower affinity compared to other antibodies.

The final concentration of PK applied to samples significantly influenced the appearance of VPSPr fragments across PRNP genotypes. Consistent with previous reports, we found VPSPr129-MM PrPSc to be the most resistant to protease K digestion, VPSPr129-VV the most sensitive, and VPSPr129-MV an intermediate between the two^10,20^. The 4 kDa fragment in the VPSPr129-VV case increased in intensity as PK concentrations increased, while the other fragments were barely detectable, even at low PK concentrations. The 4 kDa fragment of the VPSPr129-MV case behaved similarly to the VV genotype; however, unlike VPSPr129-VV, the higher molecular weight fragments were generally more prominent than the 4 kDa fragment, and these fragments migrated to slightly lower molecular weights as PK increased, presumably from fragment truncation. The VPSPr129-MM case presented unique characteristics, with the signal intensity of the 4 kDa fragment seemingly unaffected by increasing PK concentrations and the higher unglycosylated fragment decreasing slightly in intensity as PK intensified, but with no apparent truncation in response to increasing PK concentrations. Lastly, the monoglycosylated fragment in VPSPr129-MM shifted to a lower molecular weight, indicating truncation. Interestingly, each band seemed to exhibit independent PK sensitivities within this genotype.

Using an epitope mapping protocol adapted to the CE system, we characterized the population of PrP^Sc^ fragments present following PK digestion of brain tissue from confirmed cases of VPSPr. We found that for all genotypes of VPSPr, PK-resistant fragments that were reactive to 1E4 were also reactive with the type-2 CJD specific Tohoku-2 antibody. While both of these antibodies recognize overlapping PrP epitopes (Fig. 5), only Tohoku-2 recognizes the specific N-terminal PK-cleavage site exposed in type-2 CJD. Therefore, this study demonstrates that PK-resistant PrP^Sc^ fragments isolated from all genotypes of VPSPr patients at least in-part resemble those fragments found in type-2 CJD.

**Fig. 9:**
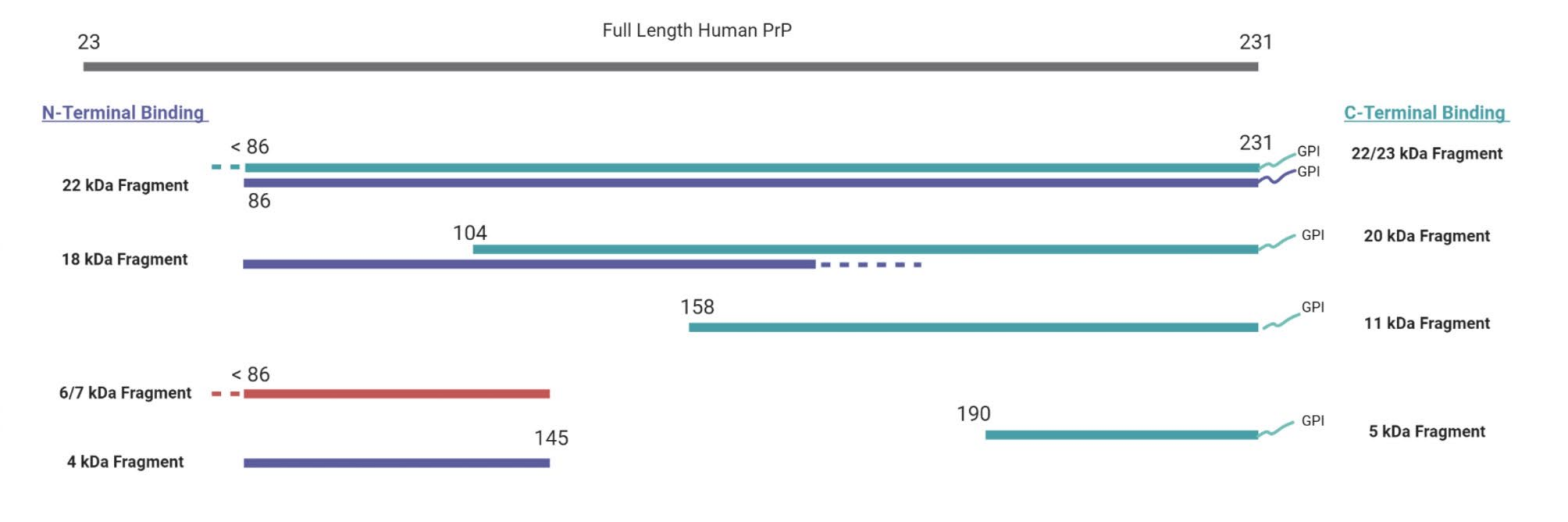
Schematic Diagram of VPSPr-Associated PK-resistant PrPSc Fragments, Adapted from Zhang et al^21^. The gray bar at the top represents full length human PrP (23–231). The purple bars represent fragments bound to N-terminal antibody 1E4 (96–105), the green bars represent fragments bound to C-terminal antibody EP1802Y (217–226), and the red fragment represents the fragment exclusively bound to N-terminal antibody 12B2 (89–93). VPSPr fragments with known cleavage sites are labelled with an amino acid cleavage site number^21^, whereas dotted lines represent the regions of currently unknown cleavage sites.

Perhaps the most surprising finding from this research is the detection of a novel VPSPr fragment which exclusively bound to antibody 12B2^21^. Interestingly, the 4 kDa band observed with all other antibodies (except for EP1802Y) was replaced with a slightly higher fragment at 6 kDa when probing with 12B2. When we exposed this fragment to increasing PK concentrations, the fragment showed sensitivity to PK and was barely or un-detectable at final PK concentrations of over 25 µg/ml. Furthermore, in VPSPr129-VV and –MV, the rate at which the 4 kDa fragment (bound to 1E4) increased in intensity as PK concentrations increased was roughly inverse to the rate at which the 6 kDa fragment (bound to 12B2) disappeared. However, this trend was not observed with VPSPr129-MM, which further emphasizes the unique features of PrP^Sc^ from VPSPr129-MM cases. Based on this data, we propose this 6 kDa fragment may be a precursor to the 4 kDa fragment prior to truncation. The generation of VPSPr fragments has previously been linked to differences in C-terminal truncation from PK^21^, yet our findings suggest additional truncation N-terminal to amino acid 86 also occurs, as the N-terminal 12B2 epitope is lost as PK concentrations intensify. To validate this theory, SAF70 (residues 156–161) was used to probe the C-terminal region adjacent to the 4 kDa cleavage site (residue 145) of VPSPr PrP^Sc^ (data not shown). There was no detection of the 6 kDa fragment regardless of PK concentration, thus supporting the N-terminal extension in the 6 kDa VPSPr fragment. To our knowledge, this is the first report of a precursor to the lowest VPSPr fragment, and it is only detectable with antibody 12B2. While 12B2 has previously been applied to VPSPr^21,22^, it is possible that slight differences at the extreme end of the molecular weight range may not be detectable using standard western blotting protocols, or due to the extreme PK-sensitivity of these fragments, western blotting may lack adequate sensitivity for detection^38^. Based on these results, we generated an updated diagram of the proposed PK-resistant fragments of VPSPr, building on the work conducted by Zhang et al.^21^, Fig. 9.

Taken together, this study provides insights into the utility of using the CE assay for analyzing VPSPr PrP^Sc^. This system provides an automated method for the reliable detection of VPSPr fragments, particularly those with very low abundances. While additional experiments are necessary to validate this assay as a robust diagnostic tool, the results presented here can serve as preliminary data to support further experiments using the CE system to analyze VPSPr heterogeneity and molecular mechanisms.

## Methods and materials

### Samples and ethics approval

Confirmed VPSPr cases were sampled from the United States, United Kingdom, and Canada. The specimens were collected from frozen brain tissue with the appropriate consent for use in research (Health Canada-Public Health Agency of Canada Research Ethics Board reference numbers REB 2009-036P and REB 2017-009P). All UK human tissue samples examined in this study were provided by the National CJD Research & Surveillance Unit (NCJDRSU), which is part of the MRC Edinburgh Brain & Tissue Bank (Edinburgh Brain Bank 16-ES-0084).

### Tissue preparation

100 mg of brain tissue was placed in an OMNI International™ Biomasher filter tube and homogenized using a disposable homogenizer column and pestle. The column was centrifuged at 14,500 rpm for 30 s to filter out solid particulate. Following the removal of the filter of the column, 900µls of homogenization buffer (100 mM NaCl, 10 mM EDTA, 0.5% NP-40, 0.5% sodium deoxycholate, 100 mM Tris-HCl, pH 8.0) was added to the tissue to yield a 10% brain homogenate. The homogenate was vortexed for one minute and centrifuged at 15,000xg for 10 min. The supernatant was removed and stored at −80 °C.

### Antibodies

The following monoclonal antibodies were used: 3F4 which recognizes residues 106–112 (dilution 1:20)^39^, 1E4 which recognizes residues 96–105 (dilution 1:20)^40^, 12B2 which recognizes residues 89–93 (dilution 1:20)^41^, 8H4 which recognizes residues 145–180 (dilution 1:10)^42^, EP1802Y which recognizes residues 217–226 (dilution 1:20)^43^ and the antibody Tohoku-2, which was graciously gifted to our lab by Dr. Tetsuyuki Kitamoto from the University of Tohoku, this antibody binds to human PrP residues 97–103 (dilution 1:10 dilution)^27^ .

### PTA precipitation, quantification, and PK digestion

Prion proteins were precipitated with sodium phosphotungstate anion (NaPTA) as described^44,45^, with modifications. Briefly, 10% brain homogenate is combined with benzonase (Millipore) at a final concentration of 0.5% and sarkosyl at a final concentration of 2%. The solution was incubated at 37 °C, shaking at 850 rpm for 2 h. 10% PTA was prepared in 18Ω water and adjusted to a pH of 7.0 with NaOH. NaPTA was added to the brain homogenate at a final concentration of 2% (v/v) and returned to incubate under the same conditions previously stated overnight. Next, the sample was centrifuged at 13,200 x g for 30 min. The supernatant was removed and the pellet was resuspended in 2% sarksoyl and 2% NaPTA. The sample was then incubated at 37 °C shaking at 850 rpm for 1 h, followed by centrifugation for 30 min at 13,200 × g. Finally, the supernatant was removed, and the pellet was resuspended in 1X DPBS. PTA-precipitated prion proteins were quantified using Invitrogen’s Qubit Fluorimeter assay. Prion proteins were diluted to a final concentration of 1.25 µg/µL in DPBS. Samples were then digested with varying Proteinase K (PK) concentrations for 1 h of shaking at 850 rpm. Protease digestion activity was stopped using EDTA-free protease inhibitor cocktail tablets (Roche). For samples that were subject to PNGase treatment, N-linked glycans were removed using the peptide-N-glycosidase F (PNGase F) kit (New England Biolabs) according to the manufacturer’s instructions.

### Capillary Western assay

Samples were analysed using the JESS capillary electrophoresis assay (ProteinSimple) as previously described^19^. Briefly, digested PTA precipitated homogenates were denatured with the ProteinSimple 5X Fluorescent Master Mix (final concentration of 1% SDS) at 95 °C for 5 min with agitation at 1000 rpm. To prepare the JESS electrophoresis plate, 5µls of prepared ladder solution and 3µls of denatured sample were loaded in row A. Rows B-E were filled with Antibody Diluent, Primary antibody, Streptavidin-HRP for detection of the ladder, Secondary antibody, and Luminol-Peroxide solution for chemiluminescent detection. Each plate can load 12 or 24 samples, depending on the Separation Module ordered. The loaded plates were centrifuged at 1000 x g for 5 min to eliminate bubbles from the wells. Plates and capillaries (2–40 kDa) were loaded into the JESS equipment, and the appropriate protocol was selected using Compass for SW software. The running time of the JESS takes approximately 3 h. Data collected by Compass for SW is presented as chemiluminescent arbitrary units vs. apparent molecular weight (MW).

### Real-time quaking-induced conversion assay (RT-QuIC)

10% (w/v) brain homogenate was serially diluted 9-fold in PBS enhanced with 0.04% (w/v) SDS and 0.4% (w/v) N2 supplement (Gibco). Reactions were prepared in a 96-well plate by combining 2 µl of diluted brain homogenate with 98 µl of prepared master mix for reactions with final concentrations of 1mM EDTA, 10nM ThT, 0.002% (w/v) SDS and 10 µg of hamster rPrP substrate (residues 23–231). The substrate was prepared in-house using recombinant expression in *Escherichia coli*, followed by purification with histidine affinity chromatography^46^. Brain homogenate dilutions, 10^− 4^ to 10^− 9^, were used to seed RT-QuIC reactions with 4 replicates per dilution for each sample. Brain homogenate from hamster 263 K and the PBS (mock)--infected hamsters were included for each RT-QuIC reaction plate as positive and negative technical controls, respectively. Reaction plates were sealed with optical adhesive film and then ran on FLUOstar Omega microplate readers (BMG). ThT fluorescence (450 nm excitation and 480 nm emission) was measured in 15-minute cycles of double-orbital shaking for 50 h. The threshold for ThT amplification was calculated as follows:

Average [Absorbance reading at 30 minutes, 60 minutes, and 1 hour 30 minutes] + 5 x Standard Deviation [Reading at 30 minutes, 60 minutes, and 1 hour 30 minutes]

Data was analyzed using Microsoft Excel (2016) and visualized on GraphPad (Version 9.5.1).

## Data Availability

All data analysis for this study is included in the manuscript. Raw RT-QuIC data or electrophoretograms of VPSPr cases are available from the corresponding author upon reasonable request.
